# Correlation between Cognitive Function and Time‐Series Variation in Eyelid Opening and Closing from Facial Videos During Neuropsychological Tests

**DOI:** 10.1002/alz.088223

**Published:** 2025-01-03

**Authors:** Terumi Umematsu, Akito Tsugawa, Naoto Takenoshita, Tomohiko Sato, Soichiro Shimizu

**Affiliations:** ^1^ NEC Corporation, Tokyo Japan; ^2^ Tokyo Medical University, Tokyo Japan

## Abstract

**Background:**

The application of image recognition technology has been spreading to dementia screening. However, cognitive function fluctuates due to mental and physical conditions. Therefore, we believe that it may be necessary to evaluate facial information over time after considering these factors. This study evaluated the correlation between cognitive function and features acquired from facial videos during neuropsychological tests.

**Method:**

Outpatients who complained of memory loss from a single memory clinic between March 2022 and April 2023 were recruited in the study. All patients underwent standard clinical tests, and facial videos were obtained during an approximately 1‐hour neuropsychological test. Cognitive function was evaluated by Mini‐Mental State Examination (MMSE). Facial feature points were calculated from the videos, then facial pose, gaze, and time‐series variation in eyelid opening and closing, such as the percentage of eyelid closure (PERCLOS), blink‐movement speed, number of blinks, eyelid time variability, and eyelid left‐right variability, were analyzed to evaluate their relationship to MMSE using the Pearson’s correlation coefficients. During neuropsychological tests, under concentration conditions, we evaluate a relationship between MMSE and the facial analysis results, particularly time‐series data of eyelid opening and closing.

**Result:**

A total of 91 patients (31 males and 60 females, age: 80.5±5.7 years, years of education: 12.8±2.7years, MMSE: 22.2±4.9 points) were included in the study. The results showed a correlation between MMSE and the time‐series variation in eyelid opening and closing, with a correlation of 0.17 with PERCLOS, 0.26 with blink‐movement speed, and 0.15 with blink counts. The correlation coefficient between blink‐movement speed and MMSE was significant, with a 95% confidence interval.

**Conclusion:**

This study showed a weak correlation between MMSE and some features of eyelid opening and closing. These results suggest that, during neuropsychological tests, when subjects are considered to be concentrating on their answers, i.e., not drowsy, an increase in the PERCLOS may be seen as one of the physical responses due to cognitive function decline.

